# Cross-Sectional Assessment of the Association of Eosinophilia with Intestinal Parasitic Infection in U.S.-Bound Refugees in Thailand: Prevalent, Age Dependent, but of Limited Clinical Utility

**DOI:** 10.4269/ajtmh.21-0853

**Published:** 2022-03-07

**Authors:** Jessica L. Webster, William M. Stauffer, Tarissa Mitchell, Deborah Lee, Elise M. O’Connell, Michelle Weinberg, Thomas B. Nutman, Potsawin Sakulrak, Dilok Tongsukh, Christina R. Phares

**Affiliations:** ^1^ Division of Global Migration and Quarantine, Centers for Disease Control and Prevention, Atlanta, Georgia;; ^2^ Department of Epidemiology and Biostatistics, Dornsife School of Public Health, Drexel University, Philadelphia, Pennsylvania;; ^3^ Department of Medicine, Center for Global Health and Social Responsibility, University of Minnesota, Minneapolis, Minnesota;; ^4^ Laboratory of Parasitic Diseases, National Institutes of Health, Bethesda, Maryland;; ^5^ International Organization for Migration, Mae Sot, Thailand

## Abstract

The most common causes of eosinophilia globally are helminth parasites. Refugees from high endemic areas are at increased risk of infection compared with the general U.S. population. It is widely accepted that eosinophilia is a good marker for helminth infection in this population, yet its absence has little predictive value for excluding infection. During an enhanced premigration health program, the CDC offered voluntary testing and management of intestinal parasites, among other conditions, to U.S.-bound refugees in Thailand. Stool specimens were tested for *Ascaris lumbricoides*, *Strongyloides stercoralis*, *Trichuris trichiura*, hookworms, *Giardia lamblia*, *Cryptosporidium* spp., and *Entamoeba histolytica* using quantitative polymerase chain reaction. Complete blood counts were performed to identify eosinophilia. Predictive values of eosinophilia for parasitic infections were calculated within nematode groups. Between July 9, 2012 and November 29, 2013, 2,004 participants were enrolled. About 73% were infected with at least one parasite. The overall median eosinophil count was 483 cells/μL (interquartile range [IQR] = 235–876 cells/μL). Compared with participants who did not test positive for any infection, higher eosinophil counts were observed in those infected with *A. lumbricoides* (RR = 1.3, 95% CI = 1.1–1.4), *S. stercoralis* (RR = 1.8, 95% CI = 1.4–2.4), *Necator americanus* (RR = 1.2, 95% CI = 1.1–1.4), and *Ancylostoma ceylanicum* (RR = 1.8, 95% CI = 1.5–2.2). Eosinophil counts were higher in younger participants (2–4 years versus 65+ years: RR = 4.2, 95% CI = 2.5–6.9), and lower in female participants (RR = 0.9, 95% CI = 0.8–0.9). Sensitivities ranged from 51% to 73%, specificities from 48% to 65%, and predictive values from 4% to 98%. The predictive value of eosinophilia is poor for the most common parasitic infections, and it should not be used alone for screening refugees.

## INTRODUCTION

Eosinophilia is frequently defined as a count of ≥ 400–500 eosinophils per μL of blood (absolute eosinophilia), or alternatively, as > 7% eosinophils on the white blood count differential.^1^ Although many conditions are associated with increased eosinophil count, such as allergic disorders, autoimmune diseases, malignancies, adrenal insufficiency, and other conditions, the most common causes globally are helminth parasites with a tissue invasive stage.^1^ The most common parasites worldwide are soil-transmitted helminths (STH) and other intestinal parasites (e.g., *Giardia lamblia*), found most commonly in areas where sanitation is poor.^2^ The WHO estimates that more than 1.5 billion people are infected with STH—approximately 24% of the world’s population.^3^

According to the United Nations High Commissioner for Refugees, over 70 million people have been forcibly displaced worldwide—the highest number in human history—with nearly 26 million of these individuals classified as refugees.^4^ Since 1975, the United States has resettled almost 3.5 million refugees through the U.S. Refugee Resettlement Program, with over 22,000 arriving in 2018.^4^ Refugees planning to move to the United States are at increased risk of parasite infection, driven by factors such as geographic origin, forced migration, age, living conditions, dietary habits, educational level, occupation, and access to sanitation, potable water, and footwear.^5^ In refugees, intestinal nematodes (*Ascaris lumbricoides*, *Trichuris trichiura*, and hookworm species) and *Strongyloides stercoralis* have been reported as the most common infections associated with eosinophilia.^6^ In clinical practice, it is widely accepted that eosinophilia is a good marker for the presence of a helminth infection in this population. However, the absence of eosinophilia has little predictive value for excluding a helminth infection. In reality, the relationship of eosinophilia to helminth infection is complex and its presence or absence in an at-risk patient must be interpreted carefully.^7^^–^^9^

There are limited published data in large cohorts describing the relationship between eosinophilia and STH, and addressing test characteristics for eosinophilia such as sensitivity, specificity, and predictive value. Understanding this association is particularly important for the most commonly encountered STH, particularly *S. stercoralis*, because of the potentially serious consequences of undetected infection.^10^

U.S.-bound refugees receive up to three scheduled medical examinations during resettlement (two overseas exams before departure for the United States, and one voluntary domestic exam after arrival). Details of these examinations are described elsewhere.^11^ CDC provides guidance for the overseas medical process, often carried out by the International Organization for Migration (IOM). Beginning in 2012, CDC, in partnership with IOM, implemented an evaluation of an enhanced overseas program to identify and manage medical conditions common in refugees—including infections with STH and other intestinal parasites—before departure for the United States. The overall timing and health intervention process, project design, and results are reported elsewhere.^11^ Here, we describe the manifestation of eosinophilia in relation to these most common parasitic infections through a sub-analysis that expands on a previously published study.^11^ In this analysis, we explore data from tests performed before antiparasitic treatment, from a cohort of refugees migrating to the United States from Thailand.

## MATERIALS AND METHODS

As part of an enhanced premigration health program, CDC personnel offered voluntary testing and management of anemia, hepatitis B virus infection, and intestinal parasites to U.S.-bound refugees, predominately Karen, living in three camps on the Thailand-Burma border. Infants under 6 months of age were excluded from this program. Written consent was obtained from participants ≥ 15 years of age and from parents or guardians of those < 15 years of age. The enhanced program included presumptive treatment of STH with albendazole and ivermectin at the initial medical examination and again at the predeparture “fit-to-fly” examination, usually conducted 3–5 days before departure. Stool and blood samples were collected at the initial medical examination (before administration of anthelmintics). Stool specimens were tested for nematodes (*A. lumbricoides*, *S. stercoralis*, *T. trichiura*, hookworms [*Necator americanus* and *Ancylostoma ceylanicum*]), *G. lamblia*, *Cryptosporidium* spp., and *Entamoeba histolytica* using a previously described quantitative polymerase chain reaction (qPCR) method,^11^ validated and developed for use in resource-limited at-risk populations.^12^^,^^13^ In this analysis, parasitic infection was defined as qPCR positive for any of these parasites. Complete blood counts were performed to identify eosinophilia. A cutoff of 400 cells/μL was used to determine eosinophilia, because the pretest probability of an elevated eosinophil count being associated with parasitic infection in this population was high, and we wanted to increase sensitivity.^2^ An analysis of the effect of treatment was previously published as part of a comprehensive description of the range of health interventions offered to this population;^11^ this evaluation provides a detailed look into the cross-sectional classification of eosinophilia in a large population before anthelmintic presumptive treatment, enabling a granular description of the association between eosinophilia and the parasites of interest.

Bivariate analyses were conducted to determine the prevalence of parasite infection within age groups and by sex, and median eosinophil count was calculated for those with and without infections, by age group and by sex. χ^2^, Wilcoxon rank sum tests, and Kruskal–Wallis rank sum tests were used to compare groups. Expected mean eosinophil counts were calculated within each age group and for each parasite using an adjusted Poisson regression model, controlling for additional detected parasite infections. These estimations were considered “expected” means, as they were the predicted outcomes based on specific parameters calculated by the model. Because each calculation controlled for each additional detected parasite infection, these estimates were interpreted as the expected mean eosinophil count for a participant with a single infection. Poisson regression was also used to calculate adjusted rate ratios (RR) and 95% confidence intervals (CI) to assess factors associated with eosinophil count. Even though some parasites were not expected to cause an elevated absolute eosinophil count (such as the protozoa, *Trichuris*), all pathogens tested were included in the bivariate and multivariate analyses as a form of validation. Sensitivity, specificity, and predictive values of eosinophilia in predicting parasitic infections were calculated within nematode groups, across three different cutoffs for eosinophilia (300, 400, and 500 cells/μL). Protozoa were excluded from the analyses of predictive values because of a known lack of association between infection and eosinophilia. Because of a small number of infected participants, *Cryptosporidium* spp. was excluded from all bivariate and multivariate analyses.

This project was reviewed in accordance with CDC institutional review policies and procedures and was determined to be non-research program implementation and evaluation.

## RESULTS

Between July 9, 2012 and November 29, 2013, 3,419 U.S.-bound refugees were offered enrollment in the enhanced predeparture health program. Of these, 2,004 (57%) participants were enrolled, and of those, blood and stool sample results were available for 1,835 (92%). The majority (44%) of participants were between 18 and 45 years old, and slightly over half (52%) were male. Out of all participants, 73% were infected with at least one of the parasites tested based on stool qPCR results, either with a single organism (32%), or with multiple organisms (41%). The most common infection was by *A. lumbricoides* (39%), followed by *T. trichiura* (32%), *N. americanus* (26%), and *G. lamblia* (22%). The overall median eosinophil count was 483 cells/μL (interquartile range [IQR] = 235–876 cells/μL). Complete demographic characteristics are shown in Table 1.

**Table 1:** Study population demographics and parasite infection status at initial medical examination (*N* = 1,835)

Age group (years)		Count (%)
< 2		68 (4)
2–4		143 (8)
5–11		337 (18)
12–17		244 (13)
18–45		813 (44)
46–64		181 (10)
65+		49 (3)
Sex		
M		946 (52)
F		889 (48)
Parasite infection by stool qPCR	*Any*	*Single*
*Ascaris lumbricoides*	726 (39)	130 (7)
*Strongyloides stercoralis*	66 (4)	13 (0.7)
*Trichuris trichiura*	598 (32)	87 (5)
*Necator americanus*	484 (26)	95 (5)
*Cryptosporidium* spp.	2 (0.1)	1 (0.1)
*Entamoeba histolytica*	94 (5)	12 (0.7)
*Giardia lamblia*	403 (22)	72 (4)
*Ancylostoma ceylanicum*	89 (5)	17 (0.9)
Number of infections*		
Multiple		756 (41)
Single		579 (32)
None		500 (27)
Eosinophil count (cells/μL)		
Median (IQR)		483 (235–876)
< 400		793 (43)
≥ 400†		1,042 (57)

IQR = interquartile range; qPCR = quantitative polymerase chain reaction. Multiple infections = detection of more than one pathogenic organism in stool by qPCR; single infection = detection of only one pathogenic organism in stool by qPCR.

* Testing was performed for *A. lumbricoides*, *S. stercoralis*, *T. trichiura*, *N. americanus*, *A. ceylanicum*, *G. lamblia*, *Cryptosporidium* spp., and *E. histolytica*.

† 400 cells/μL is the defined cutoff for eosinophilia.

Bivariate analysis revealed significant differences between age groups, sex, and eosinophil counts among those with any parasitic infection detected on stool qPCR compared with those without an infection detected (Table 2). Male participants were statistically more likely to be infected than female participants (77% versus 68%; *P* < 0.001). Median eosinophil counts varied between parasite infection status, sex, and age group. Participants who tested positive for at least one parasite had a higher median eosinophil count (532 cells/μL [IQR = 282–975]) than those without any infection detected (328 cells/μL [IQR = 160–629]). Overall, male participants had higher median eosinophil counts than female participants (571 [IQR = 300–990] versus 378 [IQR = 192–733]). When stratified by age, these differences in median eosinophil counts by sex remained statistically significant within the 5–11, 12–17, and 18–45 years old age groups (Table 3).

**Table 2:** Bivariate analysis of demographic variables by parasite infection status (*N* = 1,835)

Variable	Parasite infection*						*P* value
	Yes (*N* = 1,335)			No (*N* = 500)			
	Count	Row %	Column %	Count	Row %	Column %	
Age group (years)				
< 2	31	46	2	37	54	7	< 0.001‡
2–4	101	71	8	42	29	8	
5–11	264	78	20	73	22	15	
12–17	198	81	15	46	19	9	
18–45	583	72	44	230	28	46	
46–64	123	68	9	58	32	12	
65+	35	71	3	14	29	3	
Sex						
M	730	77	55	216	23	43	< 0.001‡
F	605	68	45	605	32	57	
Eosinophil count (cells/μL)							
Median (IQR)	532 (282–975)			328 (160–629)			< 0.001§
< 400	507	64	38	286	36	57	< 0.001‡
≥ 400†	828	79	62	214	21	43	

IQR = interquartile range.

* Any infection detected, including both single and multiple infections. Testing was performed for *A. lumbricoides*, *S. stercoralis*, *T. trichiura*, *N. americanus*, *A. ceylanicum*, *G. lamblia*, *Cryptosporidium* spp., and *E. histolytica*

† 400 cells/μL is the defined cut-off for eosinophilia.

‡ Analyzed using χ^2^ test.

§ Analyzed using Wilcoxon rank sum test.

**Table 3:** Bivariate analysis of demographic variables by eosinophil count (*N* = 1,835)

Variable	Eosinophil count*		
	Median (IQR)		*P* value
Age group (years)			
< 2	649 (430–948)		< 0.001‡
2–4	1,010 (575–1,790)		
5–11	771 (460–1,340)		
12–17	489 (268–865)		
18–45	348 (178–644)		
46–64	360 (189–554)		
65+	286 (173–412)		
Sex			
M	571 (300–990)		< 0.001§
F	378 (192–733)		
Parasite infection†			
Yes	532 (282–975)		< 0.001§
No	328 (160–629)		
Age group (years)	Male	Female	
< 2	629 (430–1,060)	684 (463–888)	0.985§
2–4	1,050 (700–1,770)	922 (511–1,840)	0.134§
5–11	937 (612–1,480)	658 (349–971)	< 0.001§
12–17	620 (357–1,000)	358 (192–645)	< 0.001§
18–45	445 (226–703)	263 (148–531)	< 0.001§
46–64	396 (234–652)	328 (146–540)	0.079§
65+	293 (167–389)	286 (186–470)	0.737§

* Eosinophil count (cells/μL).

† Any infection detected, including both single and multiple infections. Testing was performed for *A. lumbricoides*, *S. stercoralis*, *T. trichiura*, *N. americanus*, *A. ceylanicum*, *G. lamblia*, *Cryptosporidium* spp., and *E. histolytica*.

‡ Analyzed using Kruskal–Wallis rank sum test.

§ Analyzed using Wilcoxon rank sum test.

**Figure 1.**
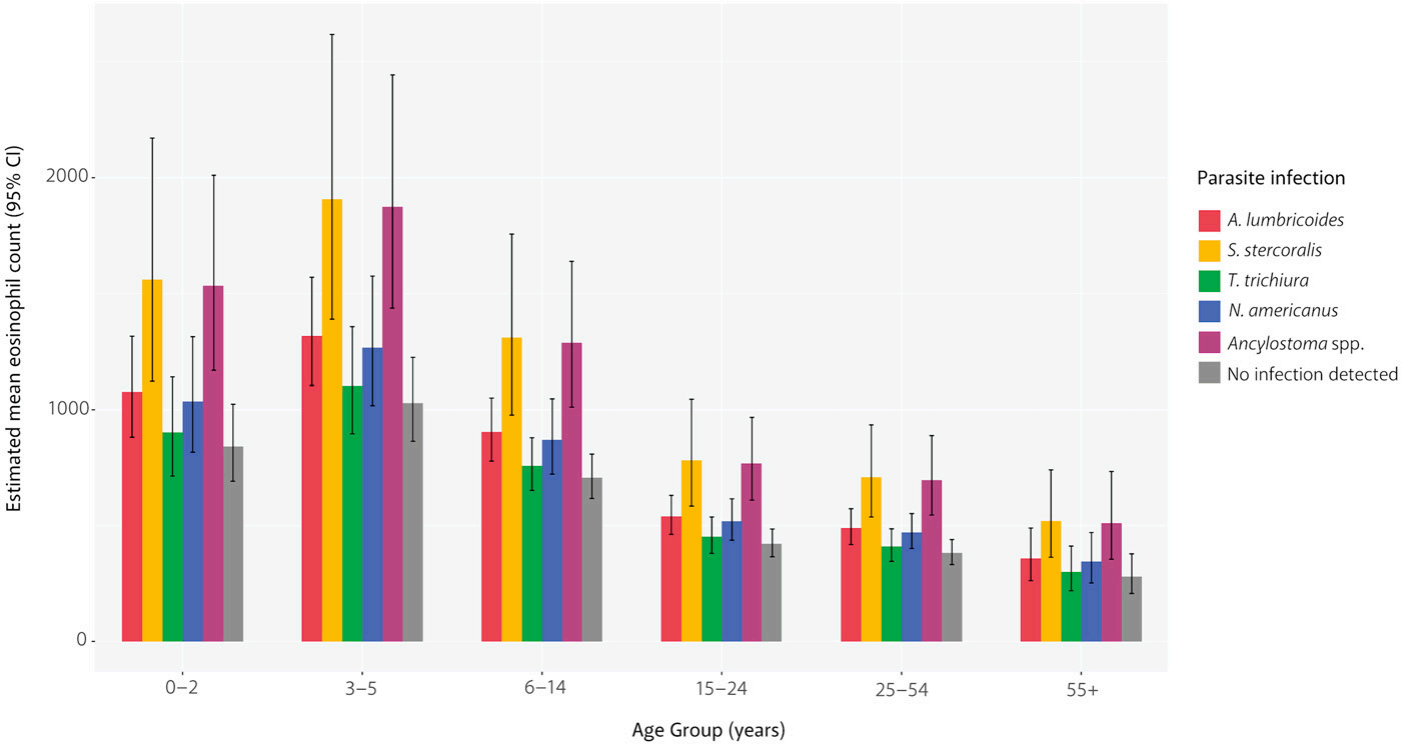
Expected mean eosinophil counts (cells/µL) of study participants by parasite infection and age group.

Overall, expected mean eosinophil counts peaked in the 2–4 years old age group and then declined as age increased. Using *A. lumbricoides* as an example, the expected mean eosinophil count was at its highest among participants aged 2–4 years with 1,339 cells/μL (95% CI = 1,136–1,577), then steadily declined to 315 cells/μL (95% CI = 194–512) among participants aged 65 years and older. This trend across age groups was consistent for all parasite infections, including in participants with no infection detected (Figure 1/Table 4). When comparing between parasites, *S. stercoralis* and *A. ceylanicum* had the highest expected mean eosinophil counts across all age groups. The expected mean eosinophil count for children in the 2–4 years old age group was 1,961 cells/μL (95% CI = 1,434–2,681) if infected with *S. stercoralis*, and 1,917 cells/μL (95% CI = 1,485–2,474) if infected with *A. ceylanicum.* This is notably higher than the expected mean eosinophil counts for those of the same age group with no infection detected (1,048 cells/μL, 95% CI = 889–1,236). However, even among participants with no infection detected, the expected mean eosinophil counts in the four youngest age groups were higher than our designated cutoff for eosinophilia of 400 cells/μL (Table 4). As anticipated, the protozoa (*E. histolytica* and *G. lamblia*) had the lowest expected mean eosinophil counts across all age groups.

**Table 4:** Expected mean eosinophil counts (cells/μL) of study participants by parasite infection and age group

	Mean estimate (95% CI) (cells/μL)							
	*A. lumbricoides*	*S. stercoralis*	*T. trichiura*	*N. americanus*	*E. histolytica*	*G. lamblia*	*A. ceylanicum*	No infection detected
Age group (years)								
< 2	928 (707–1,218)	1,359 (932–1,982)	777 (578–1,045)	896 (662–1,212)	789 (552–1,128)	743 (556–992)	1,330 (955–1,849)	727 (556–950)
2–4	1,339 (1,136–1,577)	1,961 (1,434–2,681)	1,121 (915–1,372)	1,293 (1,048–1,594)	1,138 (854–1,518)	1,071 (893–1,286)	1,917 (1,485–2,474)	1,048 (889–1,236)
5–11	1,043 (893–1,218)	1,528 (1,134–1,059)	873 (747–1,021)	1,007 (831–1,222)	887 (685–1,150)	835 (717–973)	1,494 (1,167–1,912)	817 (709–940)
12–17	606 (503–731)	888 (649–1,214)	507 (417–617)	585 (474–722)	515 (386–688)	485 (395–595)	868 (674–1,117)	474 (397–567)
18–45	497 (432–572)	728 (553–959)	416 t(356–487)	480 (413–558)	423 (326–548)	398 (336–471)	712 (566–895)	389 (344–440)
46–64	487 (384–617)	713 (519–980)	408 (320–519)	470 (374–591)	414 (300–571)	390 (303–501)	697 (517–939)	381 (306–475)
65+	315 (194–512)	461 (280–761)	264 (162–430)	304 (186–496)	268 (157–458)	252 (153–415)	451 (266–765)	247 (152–399)

Expected mean eosinophil counts were calculated within each age group and for each parasite using an adjusted Poisson regression model, controlling for additional detected parasite infections.

**Table 5:** Adjusted RR and 95% CI for estimated eosinophil count by number of infections, age group, and sex

Variable	Rate ratio (95% CI)	*P* value
Number of infections		
Single	1.19 (1.02–1.40)	0.029
Multiple	1.57 (1.36–1.82)	< 0.001
No infection detected	Ref	–
Age group (years)		
< 2	2.49 (1.45–4.25)	< 0.001
2–4	3.52 (2.16–5.73)	< 0.001
5–11	2.58 (1.60–4.16)	< 0.001
12–17	1.65 (1.01–2.69)	0.047
18–45	1.40 (0.87–2.26)	0.161
46–64	1.40 (0.84–2.33)	0.194
65+	ref	–
Sex		
M	ref	–
F	0.83 (0.74–0.93)	0.001

RR were calculated using Poisson regression models. RR in this model are comparing estimated mean eosinophil counts (cells/μL) between study participants with a single parasitic infection, or multiple parasitic infections, compared to those with no infection detected, between each age group compared to those aged 65 and older, and between females and males.

**Figure 2.**
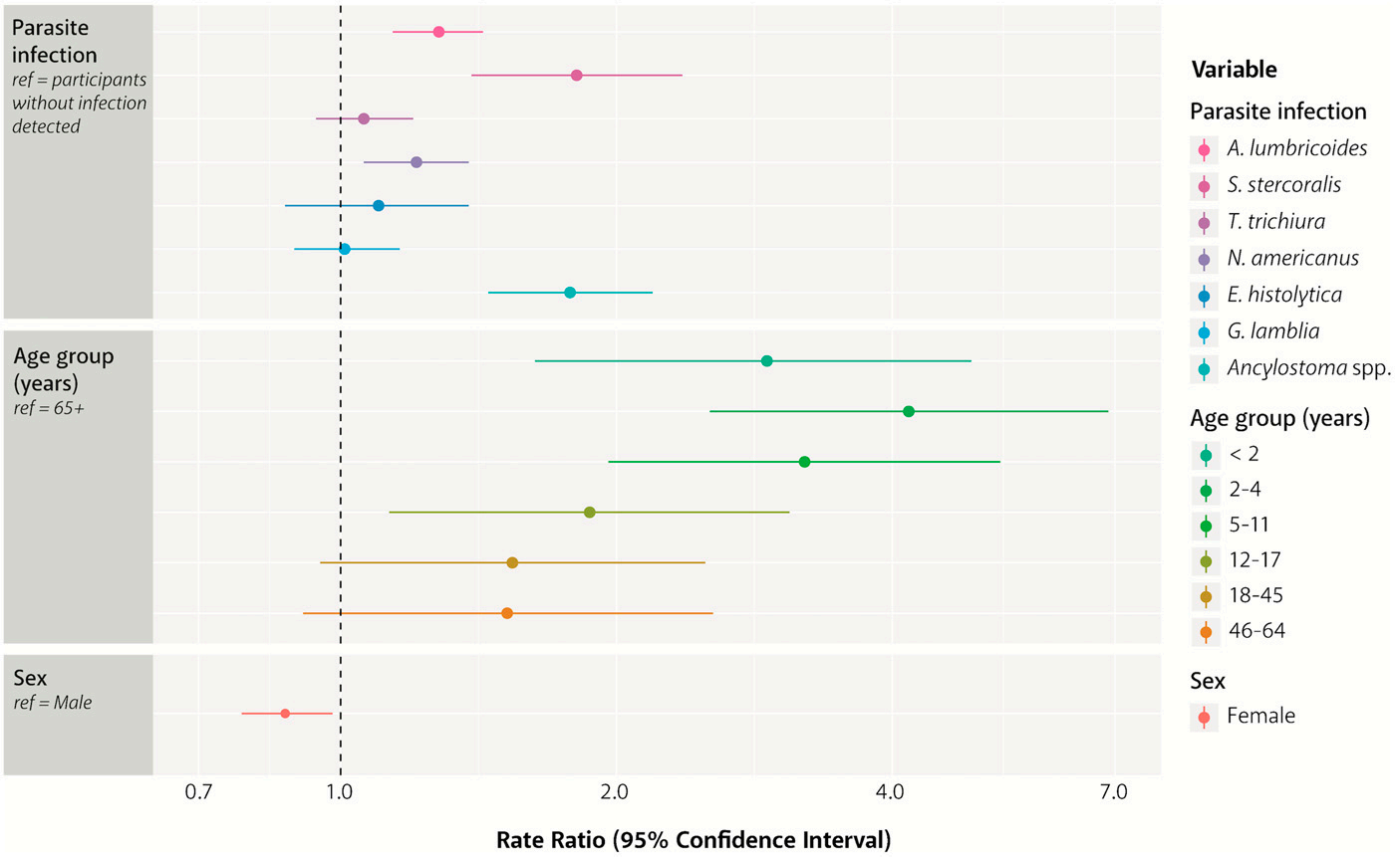
Adjusted RR and 95% CIs for eosinophil counts by parasite infection, age group, and sex.

Adjusted RR and 95% CI for eosinophil counts by parasite infection, age group, and sex are shown in  Figure 2 and Table 5. RR in this model estimated mean eosinophil counts among study participants infected with each parasite (with or without other infections) compared with those with no infection detected, between each age group and between female and male participants, adjusting for other variables listed. A significant increase in eosinophil count was seen in participants infected with *A. lumbricoides* (RR = 1.3, 95% CI = 1.1–1.4), *S. stercoralis* (RR = 1.8, 95% CI = 1.4–2.4), *N. americanus* (RR = 1.2, 95% CI = 1.1–1.4), and *A. ceylanicum* (RR = 1.8, 95% CI = 1.4–2.2) compared with those with no infection detected. Within age groups, eosinophil counts were significantly higher in the four youngest age groups when compared with the oldest age group, especially in participants aged 2–4 years, in whom the rate was over four times that of those aged 65 years and older (RR = 4.2, 95% CI = 2.5–6.9). Eosinophil counts differed by sex within this adjusted model, with female participants having significantly lower counts than male participants (RR = 0.9, 95% CI = 0.8–0.9).

Sensitivity, specificity, and predictive values of eosinophilia were calculated at varying eosinophil count cutoffs for individuals with a single infection with *A. lumbricoides*, *S. stercoralis*, *T. trichiura*, hookworms, or for those with any of the nematode infections tested (Table 6). When using a cutoff of ≥ 400 cells/μL, sensitivities ranged from 51% for any hookworm infection (*N. americanus* or *A. ceylanicum*) to 62% for *S. stercoralis* infection and for any nematode infection. Overall, sensitivities were lower when using a higher eosinophil cutoff of ≥ 500 cells/μL (ranging from 33% to 62%), and higher when using a lower cutoff of ≥ 300 cells/μL (56–73%). Specificities ranged from 48% to 65%, increasing as the cutoff increased. Positive predictive values ranged from 4% to 80%. Negative predictive values were slightly higher, ranging from 34% to 98%. Predictive values were consistent within parasite groups across cutoffs.

**Table 6:** Sensitivity, specificity, and predictive value of eosinophilia in predicting pathogenic parasitic infections*†

	≥ 300 cells/μL	≥ 400 cells/μL	≥ 500 cells/μL
*Ascaris lumbricoides*			
Sensitivity	0.69	0.60	0.52
Specificity	0.48	0.58	0.65
Positive predictive value	0.32	0.34	0.35
Negative predictive value	0.81	0.80	0.79
*Strongyloides stercoralis*			
Sensitivity	0.62	0.62	0.62
Specificity	0.48	0.58	0.65
Positive predictive value	0.04	0.05	0.06
Negative predictive value	0.97	0.98	0.98
*Trichuris trichiura*			
Sensitivity	0.56	0.46	0.33
Specificity	0.48	0.58	0.65
Positive predictive value	0.21	0.21	0.19
Negative predictive value	0.82	0.81	0.80
Hookworms (*N. americanus* and *Ancylostoma* spp.)			
Sensitivity	0.62	0.51	0.42
Specificity	0.48	0.57	0.65
Positive predictive value	0.25	0.25	0.25
Negative predictive value	0.82	0.81	0.80
Any nematode			
Sensitivity	0.73	0.62	0.53
Specificity	0.48	0.57	0.65
Positive predictive value	0.79	0.79	0.80
Negative predictive value	0.40	0.36	0.34

* Statistics calculated for persons with a single infection; persons with coinfections were excluded.

† Protozoa were excluded from the analyses of predictive values because of a known lack of association between infection and eosinophilia.

## DISCUSSION

Intestinal parasitic infection was common in this population, with 73% of refugees testing positive for at least one parasite of interest. Similar studies among individuals living in Thailand show great discrepancies in the prevalence of parasite infections by location, specifically, between rural hill-tribe and urban communities. A 2007 study on intestinal parasite infections in Thai school children revealed a prevalence of 13% in suburban Nakhon Prathom Province in Central Thailand, compared with 68% in rural Nan Province in Northern Thailand.^14^ Our findings are closer to that reported in the rural hill-tribe communities, which is not surprising given the rural locations of the camps where these refugees reside. The discrepancies in prevalence between rural and urban communities are thought to be associated with the differences in access to modern sanitation, general living conditions, and accessibility to health services.^14^^,^^15^

**Figure 3.**
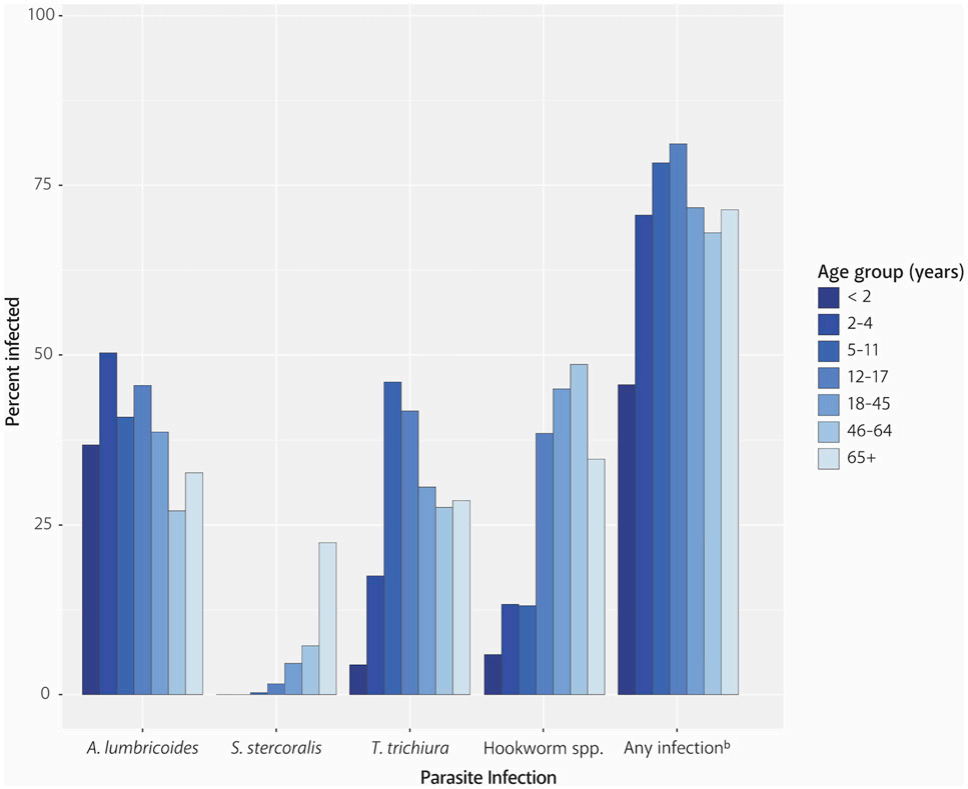
Percent of study participants infected with at least one intestinal parasite, by organism and within age groups.

Multiple infections were common, with 41% of participants testing positive for multiple infections, 32% testing positive for a single infection, and only 27% having no infection of interest. This may be an underestimation of parasitic infection in the population because participants with a single infection, or no apparent infection, could have had other parasitic infections not tested (e.g., tapeworm or fluke infections). In similar studies, the prevalence of multiple infections has ranged from 2% to 57%.^16^^–^^18^ The most common infections observed in this analysis were *A. lumbricoides* (39%), *T. trichiura* (32%), *N. americanus* (26%), and *G. lamblia* (22%). These proportions (particularly for *A. lumbricoides*) are much greater than what has previously been reported in studies based in Thailand; however, it is important to note that the majority of these studies used microscopy as opposed to qPCR to test the samples.^17^^–^^20^ Prevalence of any parasitic infection was associated with age, starting at 46% in infants aged < 2 years, peaking at 81% in children aged 12–17 years, and then steadily declining to 71% in older adults (aged 65 and older) (Figure 3). A greater proportion infected by the parasites transmitted through a fecal-oral route (*A. lumbricoides*, *T. trichiura*, and *G. lamblia*) was observed in the younger age groups, and this proportion decreased over time. This is not surprising, given an increased exposure in children due to a tendency to crawl at ground level and put their hands and objects in their mouths,^21^ coupled with the relatively short life span of these organisms (∼1–3 years). For hookworm spp. and *S. stercoralis*, an increase in prevalence was observed as age increased, also expected because the infection route is percutaneous, and these organisms have a prolonged life span in the host (5 or more years).^22^^,^^23^

The cutoff for determining eosinophilia has been loosely defined in the literature (ranging from ≥ 400 to 700 cells/μL), with the majority of reports using ≥ 500 cells/μL.^1^^,^^8^^–^^10^^,^^24^^,^^25^ Because of the high pretest probability for parasitic infection in our population, we elected to use a lower eosinophil count cutoff of ≥ 400 cells/μL to increase sensitivity. Median eosinophil count in our population was relatively high at 483 cells/μL, and more than half had eosinophilia (≥ 400 cells/μL). This proportion is consistent with previous reports, where prevalence of eosinophilia in immigrant and refugee populations ranged from 19% to 50%.^10^^,^^24^^–^^29^ Prior reports varied in their definition of eosinophilia (between 400 and 500 cells/μL), which could explain the lower prevalence estimates in some reports. Participants infected with *A. lumbricoides*, *S. stercoralis*, *N. americanus*, and *A. ceylanicum* had significantly higher eosinophil counts than those without any detectable parasite infection (Figure 2). The greatest difference was seen in refugees infected with *S. stercoralis*, with 81% higher eosinophil counts than those without any infection detected. With the exception of *Sarcocystis* and *Cystoisospora,* protozoa are not associated with eosinophilia; as might be expected, eosinophil count was not significantly higher for refugees infected with the protozoan *E. histolytica* or *G. lamblia*. In our data, *T. trichiura* was also not associated with eosinophilia, not surprisingly, because it is the only helminth with an entirely enteral lifecycle (no tissue invasion at any stage).

In addition to type of parasite infection, sex and age were significantly related to eosinophil count in our population. Overall, men and boys had higher median eosinophil counts than women and girls. This trend remained after stratifying by age, with a particularly apparent difference between the sexes in the three middle age groups (encompassing those aged 5–45 years). Previous reports attribute this relationship between sex and eosinophils to the role of hormones on immune response.^30^^,^^31^ Another explanation could be the differences in exposures between the sexes. Age alone (without the stratification by sex) was also significantly associated with eosinophil counts in our population. We observed that eosinophil counts were already elevated in children under 2 years of age, increased to peak in the 2–4 years old age group, then steadily declined as age increased. This trend was apparent across all parasite infections. Although frequently observed in clinical practice, this association between age and magnitude of eosinophil count, with and without known parasite infection, has not been well characterized to our knowledge. Children tend to have a higher baseline absolute eosinophil count due to a propensity toward a higher total leukocyte count, and may also be associated with a greater likelihood they would have a more brisk response on their first infection. There may be other, untested or yet to be described, physiologic reasons why younger children experience a more exaggerated eosinophil response. These data suggest it might be beneficial to reassess “normal” eosinophilia counts (or higher than normal eosinophil counts) in younger age groups. In our population, in adults aged 65 years and older, the only infections with a statistically significant association with eosinophilia were *S. stercoralis* and *A. ceylanicum.*

For the 1,335 participants with one or more parasitic infections of interest, eosinophilia was a poor predictor of infection. Sensitivities and specificities of eosinophilia were low across all parasites and across varying eosinophil cutoffs (ranging from 33% to 73%). Although negative predictive values were somewhat higher (ranging from 34% to 98%), a lack of eosinophilia was insufficient to rule out infection. Our data suggest that although eosinophilia should not be ignored clinically, it may not be useful to rule-in, or rule-out, helminthic infection. As described in Table 4, many noninfected participants, particularly those in the younger age groups, presented with elevated eosinophilia. This likely played a role in the observed low calculated predicted values. Other studies based on parasite detection using stool ova and parasites examination have also reported poor sensitivity and predictive value.^32^^,^^33^ Our study demonstrates that even when using qPCR, a more sensitive test than stool ova and parasites, predictive value remains low.

Our study has limitations. The main limitation is that it focused solely on the parasitic infections detected by qPCR. In this population, in particular, there are other less common parasitic infections that can be associated with eosinophilia that were not investigated, such as cestodes (e.g., tapeworm) and trematodes (e.g., *Paragonimus*, *Opisthorchis*), although prevalence of these would not be expected to exceed 2–3%.^34^ An initial test for ova and parasites by wet mount were largely negative and revealed very few additional infections not included in the qPCR results, and were not felt to be of enough value to report here.^11^ Although schistosomiasis occurs in Burma, it has not been well reported in the Northern and Eastern regions where this refugee population originates. Serologic testing for *Schistosomiasis japonicum* was performed in this cohort with < 2% being positive (unpublished data), and reports of *Schistosomiasis mekongi* have not been reported in this population to our knowledge making eosinophilia due to undetected schistosomiasis unlikely. Another limitation is the potential lack of precision due to small cell sizes, particularly in the youngest and oldest age groups. Indeed, the CI for the age-specific RR were large, however, the expected trend remained apparent despite this. Additionally, this analysis was conducted on a very specific population of refugees, though our findings are largely in concordance with analyses in other populations.

In conclusion, intestinal parasite infection and eosinophilia were both prevalent in this population, yet eosinophilia was not determined to be a strong predictor of infection. Age was highly correlated with eosinophil counts, with participants with young children having significantly higher counts than participants in the older age groups, regardless of infection status. Additionally, differences in eosinophil counts were observed between the sexes regardless of age. Our data suggest that the predictive value of eosinophilia is poor for the most common parasitic infections, and it should not be used alone for screening for these infections. However, when present, eosinophilia should not be ignored, and factors such as history, exposure, other signs or symptoms, and addition diagnostic testing may be useful to help identify an etiology.
